# Outcomes and associated risk factors of patients traced after being lost to follow-up from antiretroviral treatment in Lilongwe, Malawi

**DOI:** 10.1186/1471-2334-11-31

**Published:** 2011-01-27

**Authors:** Ralf Weigel, Mindy Hochgesang, Martin WG Brinkhof, Mina C Hosseinipour, Matt Boxshall, Eustice Mhango, Brains Nkwazi, Hannock Tweya, Maggie Kamlaka, Frederick Chagwera, Sam Phiri

**Affiliations:** 1 Lighthouse Trust at Kamuzu Central Hospital and Ministry of Health, Lilongwe, Malawi; 2 Centers for Disease Control and Prevention, Global AIDS Program, Lilongwe, Malawi (2004-2007); Maputo, Mozambique; 3 International epidemiological Databases to Evaluate AIDS (IeDEA), University of Bern, Institute of Social and Preventive Medicine, Division of International and Environmental Health, Bern, Switzerland and Swiss Paraplegic Research, Nottwil, Switzerland; 4 University of North Carolina Project Lilongwe, Private Bag A104, Lilongwe, Malawi; 5 Lighthouse Trust at Kamuzu Central Hospital, Lilongwe, Malawi; 6 Ministry of Health, Department of HIV/AIDS, Lilongwe, Malawi

## Abstract

**Background:**

Loss to follow-up is a major challenge of antiretroviral treatment (ART) programs in sub-Saharan Africa. Our objective was to a) determine true outcomes of patients lost to follow-up (LTFU) and b) identify risk factors associated with successful tracing and deaths of patients LTFU from ART in a large public sector clinic in Lilongwe, Malawi.

**Methods:**

Patients who were more than 2 weeks late according to their last ART supply and who provided a phone number or address in Lilongwe were eligible for tracing. Their outcomes were updated and risk factors for successful tracing and death were examined.

**Results:**

Of 1800 patients LTFU with consent for tracing, 724 (40%) were eligible and tracing was successful in 534 (74%): 285 (53%) were found to be alive and on ART; 32 (6%) had stopped ART; and 217 (41%) had died. Having a phone contact doubled tracing success (adjusted odds ratio, aOR = 2.1, 95% CI 1.4-3.0) and odds of identifying deaths [aOR = 1.8 (1.2-2.7)] in patients successfully traced. Mortality was higher when ART was fee-based at initiation (aOR = 2.3, 95% CI 1.1-4.7) and declined with follow-up time on ART. Limiting the analysis to patients living in Lilongwe did not change the main findings.

**Conclusion:**

Ascertainment of contact information is a prerequisite for tracing, which can reveal outcomes of a large proportion of patients LTFU. Having a phone contact number is critical for successful tracing, but further research should focus on understanding whether phone tracing is associated with any differential reporting of mortality or LTFU.

## Background

The large proportion of patients lost to follow-up (LTFU) is one of the major challenges to the ongoing success of antiretroviral treatment (ART) programs in sub-Saharan Africa [1-3]. Data from a network of ART programs in resource-limited settings showed that on average 21% of patients had been lost from programs in the first six months after starting ART [3]. A systematic review found that about 40% of patients in sub-Saharan Africa were lost at two years, with large variation in retention rates between programs [2]. A further systematic review and meta analysis of studies, which determined the vital status of patients LTFU after starting ART, found that mortality among successfully traced patients in African ART program ranges from 12% to 87%, with a combined mortality of 46% [4].

A better understanding of risk factors for tracing success and mortality among patients LTFU could help to develop targeted interventions to prevent LTFU and reduce mortality; risk factors for mortality have not been evaluated systematically [4]. ART clinics face serious operational challenges to trace patients LTFU. True outcomes of patients LTFU are difficult to assess: definitions and intervals that trigger tracing vary, active tracing of patients is labor- and time-intensive, and in 34% of traced patients the vital status still remains unknown [4]. Different tracing methods and their success rates are poorly described. Confronted with this, ART-clinics in settings with high demand for ART but with limited human resources may decide to focus on equipping patients initiating ART with good knowledge about adherence rather than investing limited resources in efforts to follow-up mobile patients in their communities [5].

In 2003, adult HIV prevalence in Malawi was estimated at 11.4% and about 240,000 people were in need of ART [6]. In response, Malawi's free national ART program started in June 2004 as a generalized public health approach [7-9]. To improve ART adherence and program retention, the national ART guidelines recommend "frontloading" patients with information on the implications of ART treatment before initiation, encouraging peer support by a family member or designated guardian, and methodical documenting patient's outcomes at every visit [10-12]. By the end of 2005 mortality and loss to follow-up (defined as patients not seen in the clinic for more than 60 days after anticipated expire of individual drug supply) in the national cohort was 10% and 8%, respectively [13].

In 2005, at the height of rapid scale-up of ART in Malawi, we set out to determine the true outcomes of patients LTFU up from ART at Lighthouse, a major provider of HIV-related services for adults and children in Malawi, and to identify risk factors for successful tracing and death.

## Methods

### Setting

The Lighthouse Trust (Lighthouse) is part of Kamuzu Central Hospital, the tertiary health facility of Malawi's central region within the capital Lilongwe and has offered HIV counseling and testing, clinical and home-based care since 2002 [14]. From 2002 to 2004, Lighthouse was the only public-sector ART clinic for the central and northern region and about 60 patients started each month on a fee-basis ("paying period"). In June 2004, the Ministry of Health opened 60 ART clinics in the public sector throughout Malawi, and Lighthouse began offering free ART, with a target of starting 150 new patients on ART per month.

### Registration, ART-initiation and follow-up

Lighthouse has maintained electronic data for all initial and follow-up visits since its inception. Details of procedures for clinic registration and ART initiation during the "paying period" have been described elsewhere [15]. In line with the 2003 national ART-guidelines, Lighthouse implemented standardized procedures for patient management and monitoring[10]. In short, HIV-infected patients register at the clinic with name, age, sex, place of residence, phone number and are asked consent to be contacted if they miss their appointment. After registration, clinicians treat active opportunistic diseases and provide cotrimoxazole prophylaxis. Patients in WHO stage 3 and 4 or with a CD4 count of below 200 cells/μl (until 2005) or below 250 cells/μl (from 2006) are eligible for ART. For children (as defined as persons 0-14 years old at the time of ART initiation) the age-dependent absolute CD4 count value thresholds for severe immunosuppression were used. The majority of children started at an age, when adult normal values are reached. To initiate ART, patients are required to attend a group education session and then are started on a fixed dose combination regimen of d4T/3TC/NVP with a two week lead-in phase. Thereafter, patients are seen monthly and receive an ART supply for 30 days. Pill counts are recorded at each visit to assess adherence. Appointments are given according the remaining pills and the new supply. After attending a refresher adherence counseling session, patients are eligible to receive a supply for 60 days and visit the clinic every 2 months. If acutely ill, patients are referred to Kamuzu Central Hospital wards. Clinic staff updates outcomes on the patient master card at every visit and the ART register is updated at least quarterly.

### Study population and data collection

Between August and December 2005 we actively traced patients LTFU, i.e. patients who were at least two weeks late for a scheduled appointment according to their last ART supply. We thus included patients with shorter delays than 60 days proposed by the Malawian national program guidelines as to evaluate the effect of earlier tracing on patient outcomes. The study population was restricted to all patients ever starting ART at the Lighthouse clinic until 31^st ^of May 2005. We verified contact information (phone and place of residence) and whether patients consented for external tracing; Lighthouse began collecting this contact information in May 2004, so patients already LTFU by this time were not traced. We excluded patients living outside Lilongwe city without a phone number, patients living within Lilongwe city but without address and phone number and those who did not consent for tracing.

Using the list of eligible patients, Kamuzu Central Hospital death registry was cross-checked for Lighthouse patients and their date of death, and the ART register at Mzuzu Central Hospital in the northern region of Malawi, which opened its ART clinic shortly before start of the study, was searched for Lighthouse patients LTFU whose transfer out was not registered.

Study assistants contacted the patients, family members or contact persons by phone or in-person. A brief survey instrument was used to collect vital status and relevant information on current ART use, current site of care, or date of death. All study data were collected on paper forms and subsequently entered into an electronic database.

### Statistical analysis

We used descriptive analyses to examine demographic and clinical characteristics and tracing history of patients LTFU and their outcomes. Outcomes were classified as patient alive, died or untraceable. Patients found alive were further categorized as on ART, transferred out to another ART program or having stopped ART.

We performed logistic regression for both successful tracing and mortality among successfully traced patients to determine risk factors for these outcomes and, in a second step, performed a sensitivity analysis restricted to patients living in Lilongwe. Predictor variables for both models included gender, age at start of ART (in years; groups 15-29; 30-39; 40-49; ≥ 50), time period of starting of ART (2 groups: until June 2004, paying for ART; June 2004 or later, free ART), reason for starting ART (4 groups: low CD4 count; WHO stage 3; WHO stage 4; other or unknown), follow-up time from start of ART until the last patient contact (time of last visit in months: no follow-up; 1-6; 7-12; 13 or later) and providing a telephone contact. We evaluated adjusted odds ratios that were mutually adjusted for all other predictor variables.

The day and month of death was commonly unknown for patients who were traced outside of the health facilities. To evaluate mortality rates since ART initiation in patients LTFU with known outcomes (i.e., those traced successfully) we performed sensitivity analysis by stepwise increasing the presumed date of death by 3 months between one day after the last contact date and the date of successful tracing for that patient. Under these different scenarios we evaluated mortality among successfully traced patients using Kaplan-Meier analysis over months 1 to 12 and months 13 to 24. Rates are expressed in deaths per 100 person-years. All analyses were done using Stata (version 10.1; Stata Corporation, College Station, Texas, USA).

### Ethical approval

The study has ethical approval from the National Health Science Research Committee (NHSRC), the national ethics committee in Malawi. General measures were provided in all ART facilities to ensure patient confidentiality, consent for HIV testing and counseling, and support for those who received a positive HIV test result. Data collected for this study did not include personal identifiers. Since the study was partly funded by the U.S. Centers for Disease Control and Prevention (CDC) and involved a co-investigator from CDC, the protocol had to undergo ethics review by CDC. As the study design and purpose aims at evaluating and improving services, it was approved and classified as program evaluation, a type of public health non-research according to CDC regulations [16].

## Results

### Recruitment of study participants, baseline characteristics and outcomes of patients lost to follow-up

**Figure 1:**
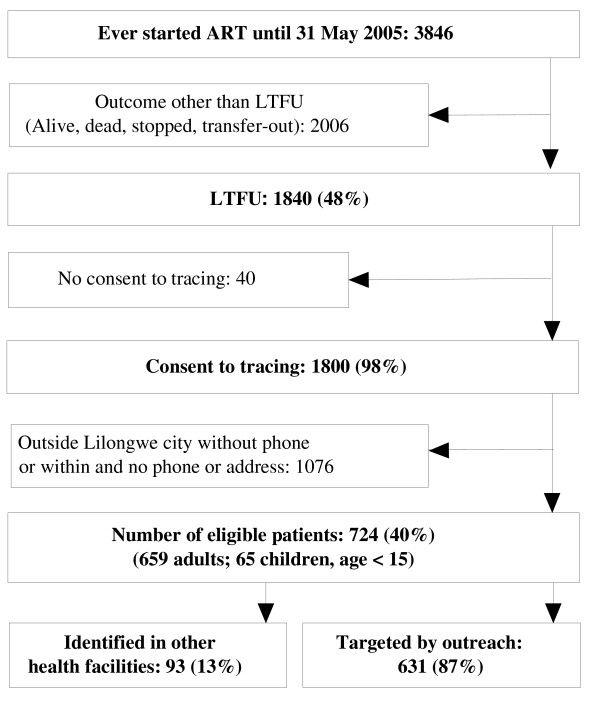
**Scheme of recruitment for the tracing study (N = 724)**.

Until 31^st ^May 2005, 3846 patients had ever started ART at the Lighthouse and 1840 (48%) were LTFU, of whom 1800 (98%) had given consent to tracing (Figure 1). For 1076 patients the contact address was outside Lilongwe and no phone contact was given or was within Lilongwe and neither phone number nor address was given. From the list of the patients remaining eligible for tracing, a review of records in Lilongwe and Mzuzu Central Hospitals revealed vital status of 93 patients, so that 631 patients had to be traced by outreach, either by phone or in-person. In total we thus evaluated outcomes of 724 patients LTFU that included 659 adults and 65 children less than 15 years of age, representing 40% (724/1800) of all patients LTFU who gave consent for tracing.

Table 1 describes the baseline demographic and clinical characteristics of the study population. Adults and children had a median age of 36 and 6 years, and the percentage of females was 53% and 43%, respectively. The median year of ART start was 2004 and start of ART was free of charge for most adults (67%) and children (80%). The median (IQR) baseline CD4 counts were 71 cells/μl (22-141) and 184 cells/μl (29-423) and the most common reason for ART start was WHO stage 3 in 67% and 89% of adults and children, respectively. The median (IQR) duration of follow-up in the ART-clinic was 73 days (14-303) and 92 days (13-210), and 67% and 69% of adults and children were LTFU within 6 months after ART start. Telephone contact was available for 47% of adults and 37% of children and 86% of adults and 89% of children were living in Lilongwe.

**Table 1:** Demographic and clinical characteristics of patients included in the tracing study (N = 724)

Parameter	Adults	Children
Number (% of total)	659 (91%)	65 (9%)
Age (median, IQR)	36 (31-43)	6 (3-10)
Number (%) of females	351 (53%)	28 (43%)
Year of ART start (median; range)	2004 (2000-2005)	2004 (2002-2005)
ART free of charge at start (after 10 June 2004)	440 (67%)	52 (80%)
CD4 at start of ART (median, IQR)*	71 (22-141)	184 (28-423)
Reason ART start		
*Low CD4 count*	*72 (11%)*	*3 (5%)*
*WHO stage 3*	*444 (67%)*	*58 (89%)*
*WHO stage 4*	*124 (19%)*	*3 (5%)*
*Transfer in*	*3 (0.5%)*	*0 (0%)*
*Not recorded*	*16 (2.5%)*	*1 (1%)*
Days of follow up on ART (median, IQR)	73 (14-303)	92 (13-210)
Number (%) by time period since start of ART		
*No follow-up*	*109 (17%)*	*13 (20%)*
*Months 1 to 6*	*331 (50%)*	*32 (49%)*
*Months 7 to 12*	*78 (12%)*	*12 (19%)*
*Months 13 or later*	*141 (21%)*	*8 (12%)*
Number (%) with telephone contact ☎	307 (47%)	24 (37%)
Residence in Lilongwe	567 (86%)	58 (89%)

* missing in 30 adults and 3 children; ☎ home, cell and/or work phone

Of the 631 patients traced by outreach, 55 (9%) patients had two and 2 patients had three successive tracing attempts. Altogether, 389 (57%) tracing attempts were performed in-person and 299 (43%) by phone. First tracing attempts were successful in 61% of the in-person attempts (204/336) and 73% (214/295) by phone attempts. Tracing success declined from 66% (418/631) to 36% (20/55) from first to second tracing attempt (data not shown).

The updated outcomes after tracing are given in Table 2. In total, of those eligible for tracing, 534 (74%) patients LTFU were successfully traced. These included 486 adults and 48 children, of whom 201 (41%) and 16 (33%) respectively, were found to be dead. Among the 317 patients found to be alive, 285 (90%) were still on ART (157 at Lighthouse and 128 at another ART clinic) and 32 (10%) had stopped ART.

**Table 2:** Success of tracing and outcomes in patients successfully traced

Outcome	Total	Adults	Children
***Overall***	724	659	65
Successfully traced	534 (74%)	486 (74%)	48 (74%)
Found to have died (% of those successfully traced )	217 (41%)	201 (41%)	16 (33%)
			

***For patients found to be alive***	317	285	32
On ART at Lighthouse (% of alive)	157 (50%)	135 (47%)	22 (69%)
Transfer-out, on ART (% of alive)	128 (40%)	127 (45%)	1 (3%)
Stopped ART (% of alive)	32 (10%)	23 (8%)	9 (28%)

### Factors associated with successful tracing in adult patients

The percentage successfully traced was 81.4% and 67.0% (P < 0.001) for patients with and without a phone contact, respectively. Controlling for gender, age, treatment period, reason for start of ART and time of the last clinic visit from ART start, having a phone number doubled the odds of successful tracing (aOR 2.07, 95% CI: 1.42-3.01, P < 0.001) (Table 3). Other factors, including reasons for start of ART (P = 0.39) and follow-up time until the last clinic visit (P = 0.94) were not associated with tracing success. After restricting the analysis to patients living in Lilongwe the results stayed mostly the same and availability of a phone number remained the major risk factor (data not shown).

**Table 3:** Risk factors for successful tracing in adults lost to follow-up. Odds ratios are derived from logistic regression; P-values using likelihood ratio tests. (N = 659)

Parameter	**% traced (95% CI**^**#**^**)**	Adjusted OR (95% CI)	P
Gender			0.34
*Female*	*73.8 (68.9-78.3)*	*1.20 (0.83-1.73)*	
*Male*	*73.7 (68.4-78.5)*	*1*	
Age group			0.11
*15-29*	*63.9 (54.7-72.4)*	*1*	
*30-39*	*74.2 (68.7-79.2)*	*1.59 (0.99-2.55)*	
*40-49*	*78.5 (72.0-84.1)*	*1.91 (1.12-3.24)*	
*≥ 50*	*76.2 (63.8-86.0)*	*1.71 (0.83-3.5)*	
Reason start ART			0.39
*Low CD4 count*	*72.2 (60.4-82.1)*	*1*	
*WHO stage 3*	*73.9 (69.5-77.9)*	*1.15 (0.64-2.04)*	
*WHO stage 4*	*71.8 (63.0-79.5)*	*1.07 (0.54-2.12)*	
*Other/unknown*	*89.5 (66.9-98.7)*	*3.38 (0.7-16.43)*	
Time of last visit*			0.94
*No follow-up*	*72.5 (63.1-80.6)*	*1*	
*Months 1 to 6*	*71.3 (66.1-76.1)*	*0.95 (0.57-1.57)*	
*Months 7 to 12*	*76.9 (66.0-85.7)*	*0.92 (0.45-1.89)*	
*Months 13 or later*	*78.7 (71.0-85.2)*	*0.77 (0.34-1.74)*	
Telephone contact			< 0.001
*None*	*67.0 (61.9-71.9)*	*1*	
*Available*	*81.4 (76.6-85.6)*	*2.07 (1.42-3.01)*	
Treatment period			0.13
*Paying*	*79.5 (73.5-84.6)*	*1.66 (0.85-3.23)*	
*Free ART*	*70.9 (66.4-75.1)*	*1*	

# binomial exact 95% confidence interval

*Follow up time from start of ART until last patient visit

### Mortality and risk factors for mortality in successfully traced adults

Mortality rates in successfully traced patients between months 1 and 12 after start of ART were 58 (95% CI: 50-67), 50 (95% CI: 43-58) and 45 (95% CI: 38-53) per 100 person-years, when allowing a maximal time between the date of last visit and the presumed death date of 3, 6 and 9 months, respectively. Between months 13 and 24 the respective mortality rates were 19 (95% CI: 12-30), 20 (95% CI: 13-31) and 27 (95% CI: 19-39) per 100 person-years.

Table 4 shows the crude percentage mortality and adjusted odds ratios of death in relation to risk factors. The percentage of deaths was 64.6% in patients with no follow-up following ART initiation as compared to 50%, 16.7% and 19.8% in those with a last visit in months 1 to 6, months 7 to 12 and months 13 or later, respectively. The corresponding adjusted odds ratios were 0.55 (95% CI: 0.31-0.96), 0.073 (95% CI: 0.03- 0.18), and 0.058 (95% CI: 0.024-0.14), indicating declining mortality with increasing follow-up time (P < 0.0001). The proportion of recorded deaths was higher among successfully traced patients with a phone contact (43.6%) as compared to those without a phone (39%; aOR 1.79, 95% CI: 1.19-2.71, P = 0.005). The adjusted odds of death were further higher among patients that initiated ART during the paying period as compared to the free period [aOR = 2.28 (1.10-4.72); P = 0.022]. Gender, age group and reason for start of ART were not strongly associated to the risk of death in successfully traced patients. The sensitivity analysis restricting to patients living in Lilongwe did not reveal major changes of the results and time of last visit since ART start and availability of a phone number remained the major risk factors for mortality among successfully traced adult patients (data not shown).

**Table 4:** Risk factors for mortality in adults lost to follow-up that were successfully traced. Odds ratios are derived from logistic regression; P-values using likelihood ratio tests. (N = 486)

Parameter	**% Died (95% CI**^**#**^**)**	Adjusted OR (95% CI)	P
Gender			0.45
*Female*	*40.2 (34.1-46.4)*	*0.85 (0.56-1.29)*	
*Male*	*42.7 (36.2-49.4)*	*1*	
Age group			0.64
*15-29*	*42.3 (31.2-54.0)*	*1*	
*30-39*	*41.0 (34.2-47.9)*	*0.99 (0.56-1.77)*	
*40-49*	*38.0 (30.2-46.3)*	*1.07 (0.57-1.98)*	
*≥ 50*	*52.1 (37.2-66.7)*	*1.57 (0.70-3.50)*	
Reason start ART			0.091
*Low CD4 count*	*28.8 (17.1-43.1)*	*1*	
*WHO stage 3*	*39.3 (34.6-45.5)*	*1.28 (0.63-2.58)*	
*WHO stage 4*	*56.2 (45.3-66.7)*	*1.99 (0.88- 4.52)*	
*Other/unknown*	*28.4 (10.3-55.9)*	*0.55 (0.15-2.03)*	
Time of last visit *			< 0.0001
*No follow-up*	*64.6 (53.0-75.0)*	*1*	
*Months 1 to 6*	*50.0 (43.4-56.6)*	*0.55 (0.31-0.96)*	
*Months 7 to 12*	*16.7 (8.29-28.5)*	*0.073 (0.030-0.18)*	
*Months 13 or later*	*19.8 (12.9-28.5)*	*0.058 (0.024-0.14)*	
Telephone contact			0.005
*None*	*39.0 (32.7-45.5)*	*1*	
*Available*	*43.6 (37.4-50.0)*	*1.79 (1.19-2.71)*	
Treatment period ^‡^			0.022
*Paying*	*30.5 (23.7-37.9)*	*2.28 (1.10-4.72)*	
*Free ART*	*47.4 (41.8-53.1)*	*1*	

# binomial exact 95% confidence interval

*Follow up time from start of ART until last patient visit

^‡^Adjusted odds ratio for treatment period is reversed from the crude odds ratio (crude OR = 0.49), due to confounding by time of last visit (for detail, see Additional file 1).

## Discussion

Dedicated tracing studies of patients LTFU are an important instrument to improve quality of care as well as outcome evaluation of ART programs in sub-Saharan Africa. In the present study in Lilongwe, Malawi, only 40% of patients LTFU who gave consent could be traced since contact information was not available for many and field tracing attempts outside Lilongwe were infeasible. However, outcomes were successfully updated in 74% of those traced. Among those successfully traced, 41% of adults and 33% of children had died, most commonly in the first six months following ART initiation. Among patients found to be alive, 92% of adults and 72% of children were still taking ART. The availability of a phone contact improved the chances of successful tracing and identifying outcomes in patients LTFU. As a result of this study, we introduced regular ascertainment of contact information for each patient at the first clinic visit, recommended patients living outside Lilongwe to register at ART clinics closer to their homes and are now able to successfully trace 85% of patients LTFU [17].

A recent systematic review and meta analysis of studies that traced patients LTFU to ascertain their vital status in sub-Saharan African ART programs, which included Lighthouse, showed that on average 46% of those traced were found to have died [4]. The review also noted the scarcity of information on risk factors for successful tracing as well as death in patients LTFU as a major limitation of existing surveys. We identified short follow-up time on ART as the strongest predictor for high risk of death in successfully traced patients. Patients with no follow-up visit or a last visit in the first 6 months showed 50% to 65% mortality as compared to less than 20% mortality in those seen last after 6 months. In Uganda, deaths of patients LTFU were examined depending on the period from their last clinic visit and rates were highest early after the last visit (mortality of 115, 42 and 21 per 100 person years after 1, 2 to 6, and after 6 months since the last visit, respectively) [18]. These findings suggest that an earlier start of active tracing of patients that missed a scheduled visit date, especially when loss to follow occurred shortly after ART initiation, may help to reduce early mortality.

Studies examining associations between baseline characteristics and tracing success or death show conflicting results making targeted interventions to prevent LTFU more difficult. While we and others did not find a strong relation between age, sex and tracing success [18], in Zambia, a higher proportion of men were untraceable [19]. Advanced disease stage at baseline measured by WHO stage in our study or pre-ART CD4 count [18] were also not associated with tracing success. With respect to identifying mortality through tracing, Zambian patients who died were older, more likely to be TB/HIV co-infected, had lower BMI, advanced WHO stages and surprisingly, had higher CD4 count [19]. In contrast, advanced WHO stages were not associated with death among our patients LTFU and low CD4 count was associated with higher mortality of patients LTFU in Uganda [18]. Additionally, an earlier analysis of our dataset showed a lower median CD4 count in untraceable patients compared to traceable patients who died, and we hypothesized that many untraceable patients might have died in line with the Uganda findings [20].

Our study demonstrated that outcomes, including deaths, of patients LTFU can be found in records of other health facilities. Where death registries are operational, such as in South Africa, conclusive evidence on vital status of patients not found by active tracing can be retrieved from record linkage using unique patient identifiers [21,22]. Therefore, improved information transfer between facilities may prevent costly and time-consuming tracing.

In our study, during first tracing attempts, phone tracing was more successful in revealing outcomes than in-field tracing. The availability of a phone contact appears an effective way of identifying true outcomes of patients LTFU and was associated with mortality. However, tracing methods may ascertain outcomes differently. On the phone, guardians may not want to admit that their patients have left the clinic, but will reveal that the patient has died. Likewise, survivors may be more likely to have changed their phone number leading to a failed phone tracing attempt, but not their home address thereby increasing the likelihood of being found alive through field tracing. Both scenarios could increase the proportion of deaths among successfully traced patients via phone compared to in-person field tracing, as observed in our study. A study from South Africa found that 46% of patients LTFU could not be contacted because of incomplete or missing contact details and highlights the difficulties of phone tracing in patients without a permanent residential address, such as sharing of one cell phone by the entire family and changing of phone numbers due to theft or expiration of the SIM card [23]. However, cell phone coverage is increasing in Malawi and positive experience using phones in patient follow-up may result in wider use [24]. Therefore, more information on advantages and limitations of different tracing methods is needed.

Our findings highlight the importance of maximizing the potential for early tracing, including soliciting of patient consent to be traced and maintaining current contact information for patients who consent to be traced. Tracing was possible in only 40% of our patients LTFU, but when attempted, the patient status was verified in 74%. Nearly all patients LTFU and found to be alive in our study were still taking ART; more than half of them in our facility. This may be partly related to the early initiation of tracing, after being 2-weeks late for appointment, in a substantial proportion of patients LTFU. Patients may accumulate leftover tablets from previous supplies without telling us, receive tablets from family members on ART or from other clinics, without our information- this needs to be explored further. As a result of the present study, the period for patients becoming eligible for tracing was extended to 3 weeks and the list of patients LTFU to be traced is now updated weekly within our routine services as to avoid unnecessary contacting of returning patients.

Our study has limitations. We traced only 40% of patients LTFU who consented. This sample may not be representative of all patients LTFU, as it includes only patients who had given consent for tracing, had provided contact information in Lilongwe or had a phone when living outside, and who were in care by May 2004 and beyond. With increasing delay between LTFU and tracing, patients or guardians may have been more difficult to contact, as contact information that can change over time was not regularly updated. Guardians may further forget details of patients' ART or may report outcomes they perceive desirable by the phone or field tracers, which could result in under-reporting of the outcome "stopping treatment". In addition, we did not analyze the period from LTFU and tracing to provide further information about the importance of this window period for tracing success and outcomes. Finally, few data on children LTFU in our sample prevented us from a separate analysis and more information is needed to examine the specific features in this age group.

## Conclusions

ART programs need to maintain current contact information of patients or guardians and should be aware of the limitations of the tracing method used. They should aim for early tracing after a missed appointment for ART pick-up, especially if patients have recently started ART, including searching hospital records for patients LTFU. These interventions may increase the proportion of successfully traceable patients to return them back to care and reduce mortality; and improve overall program evaluation.

## Competing interests

The authors declare that they have no competing interests.

## Authors' contributions

RW, MH, MCH, MB and SP designed and implemented the study. EM, MK and FC collected data. BN, HT and MB performed the data management. MB analyzed the data and contributed to data interpretation. RW wrote the manuscript with critical input from MB, MCH and MH. All authors approved the final manuscript.

## Pre-publication history

The pre-publication history for this paper can be accessed here:

http://www.biomedcentral.com/1471-2334/11/31/prepub

## Supplementary Material

Additional File 1 **Table presenting follow-up time from start of ART until last patient visit and mortality in adults lost to follow-up that were successfully traced and started ART in the paying (top part; N = 174) and free ART period (lower part; N = 312)**. The percentage of deaths declines with follow-up time in the paying (until 10^th ^June 2004) as well as the free ART period (after 10^th ^June 2004), but given follow-up time from ART start until the last visit, mortality in the paying era is consistently higher. This is reflected in the adjusted OR for comparing paying with free treatment period of 2.28 (95% CI 1.10-4.72) in Table 4. The adjusted OR is reversed to the crude OR of 0.49, because of the reversed distribution of patient numbers over the Time-of-last-visit- periods, which are associated with different rates of mortality. Click here for file
