# Supplementary material for: Outcomes and associated risk factors of patients traced after being lost to follow-up from antiretroviral treatment in Lilongwe, Malawi
Source: BMC Infect Dis. 2011 Jan 27;11:31. doi: 10.1186/1471-2334-11-31 (PMC3039578; doi:10.1186/1471-2334-11-31)
Supplement: Additional File 1 — Table presenting follow-up time from start of ART until last patient visit and mortality in adults lost to follow-up that were successfully traced and started ART in the paying (top part; N = 174) and free ART period (lower part; N = 312). The percentage of deaths declines with follow-up time in the paying (until 10th June 2004) as well as the free ART period (after 10th June 2004), but given follow-up time from ART start until the last visit, mortality in the paying era is consistently higher. This is reflected in the adjusted OR for comparing paying with free treatment period of 2.28 (95% CI 1.10-4.72) in Table 4. The adjusted OR is reversed to the crude OR of 0.49, because of the reversed distribution of patient numbers over the Time-of-last-visit- periods, which are associated with different rates of mortality. [file 1471-2334-11-31-S1.DOC]

**Additional file 1:**

| **Parameter** | **% Deceased**  **(95% CI^#^)** | **Adjusted OR**  **(95% CI)** | **P** | **Number of patients (%)** |
| --- | --- | --- | --- | --- |
| Time of last visit during  paying period | | | < 0.0001 |  |
| *No follow-up* | *71.4 (47.8-88.7)* | *1* |  | *21 (12%)* |
| *Months 1 to 6* | *69.2 (38.6-90.9)* | *0.73 (0.14-3.95)* |  | *13 (7%)* |
| *Months 7 to 12* | *24.1 (10.3-43.5)* | *0.11 (0.025-0.46)* |  | *29 (17%)* |
| *Months 13 or later* | *19.8 (12.9-28.5)* | *0.084 (0.023-0.30)* |  | *111 (64%)* |
| Time of last visit during  free period | | | < 0.0001 |  |
| *No follow-up* | *62.1 (48.4-74.5)* | *1* |  | *58 (19%)* |
| *Months 1 to 6* | *48.9 (42.1-55.6)* | *0.52 (0.28-0.86)* |  | *223 (71%)* |
| *Months 7 to 12* | *9.68 (2.04-25.8)* | *0.051 (0.013-0.20)* |  | *31 (10%)* |
| *Months 13 or later* | *no data* | *no data* |  | *no data* |

# binomial exact 95% confidence interval

Odds ratios are derived from logistic regression and adjusted for age, sex, reason for ART start and availability of telephone contact. P-values are from likelihood ratio tests.
